# Adverse postoperative outcomes in elderly patients with sarcopenia

**DOI:** 10.1186/s12877-024-05066-2

**Published:** 2024-06-27

**Authors:** Yitian Yang, Mingyang Sun, Wan-Ming Chen, Szu-Yuan Wu, Jiaqiang Zhang

**Affiliations:** 1 Department of Anesthesiology and Perioperative Medicine, Henan Provincial People’s Hospital, People’s Hospital of Zhengzhou University, Zhengzhou, China; 2 Graduate Institute of Business Administration, College of Management, Fu Jen Catholic University, Taipei, Taiwan; 3 Artificial Intelligence Development Center, Fu Jen Catholic University, Taipei, Taiwan; 4 Department of Food Nutrition and Health Biotechnology, College of Medical and Health Science, Asia University, Taichung, Taiwan; 5 Big Data Center, Lo-Hsu Medical Foundation, Lotung Poh-Ai Hospital, Yilan, Taiwan; 6 Division of Radiation Oncology, Lo-Hsu Medical Foundation, Lotung Poh-Ai Hospital, Yilan, Taiwan; 7 Department of Healthcare Administration, College of Medical and Health Science, Asia University, Taichung, Taiwan; 8 Cancer Center, Lo-Hsu Medical Foundation, Lotung Poh-Ai Hospital, Yilan, Taiwan; 9 Centers for Regional Anesthesia and Pain Medicine, Taipei Municipal Wan Fang Hospital, Taipei Medical University, Taipei, Taiwan; 10 Department of Management, College of Management, Fo Guang University, Yilan, Taiwan

**Keywords:** Sarcopenia, 30-day adverse postoperative outcomes, 90-day adverse postoperative outcomes, Surgery, Old-age

## Abstract

**Purpose:**

No study has compared 30-day and 90-day adverse postoperative outcomes between old-age patients with and those without sarcopenia.

**Patients and methods:**

We categorize elderly patients receiving major surgery into two groups according to the presence or absence of preoperative sarcopenia that were matched at a 1:4 ratio through propensity score matching (PSM). We analyzed 30-day or 90-day adverse postoperative outcomes and mortality in patients with and without sarcopenia receiving major surgery.

**Results:**

Multivariate logistic regression analyses revealed that the patients with preoperative sarcopenia were at significantly higher risk of 30-day postoperative mortality (adjusted odds ratio [aOR]. = 1.25; 95% confidence interval [CI]. = 1.03–1.52) and 30-day major complications such as postoperative pneumonia (aOR = 1.15; 95% CI = 1.00-1.40), postoperative bleeding (aOR = 2.18; 95% CI = 1.04–4.57), septicemia (aOR = 1.31; 95% CI = 1.03–1.66), and overall complications (aOR = 1.13; 95% CI = 1.00-1.46). In addition, surgical patients with sarcopenia were at significantly higher risk of 90-day postoperative mortality (aOR = 1.50; 95% CI = 1.29–1.74) and 90-day major complications such as pneumonia (aOR = 1.27; 95% CI = 1.10–1.47), postoperative bleeding (aOR = 1.90; 95% CI = 1.04–3.48), septicemia (aOR = 1.52; 95% CI = 1.28–1.82), and overall complications (aOR = 1.24; 95% CI = 1.08–1.42).

**Conclusions:**

Sarcopenia is an independent risk factor for 30-day and 90-day adverse postoperative outcomes such as pneumonia, postoperative bleeding, and septicemia and increases 30-day and 90-day postoperative mortality among patients receiving major surgery.

**Condensed abstract:**

No study has compared 30-day and 90-day adverse postoperative outcomes between patients with and those without sarcopenia. We conducted a propensity score?matched (PSM) population-based cohort study to investigate the adverse postoperative outcomes and mortality in patients undergoing major elective surgery with preoperative sarcopenia versus those without preoperative sarcopenia. We demonstrated that sarcopenia is an independent risk factor for 30-day and 90-day adverse postoperative outcomes, such as postoperative pneumonia, bleeding, septicemia, and mortality after major surgery. Therefore, surgeons and anesthesiologists should attempt to correct preoperative sarcopenia, swallowing function, and respiratory muscle training before elective surgery to reduce postoperative complications that contribute to the decrease in surgical mortality.

**Supplementary Information:**

The online version contains supplementary material available at 10.1186/s12877-024-05066-2.

## Introduction

Sarcopenia is defined as the progressive and generalized loss of skeletal muscle mass and strength with a risk of adverse outcomes, such as physical disability, poor quality of life, and even death [1]. Lifestyle, physical inactivity, malnutrition, and chronic diseases (e.g., osteoporosis and metabolic diseases) are all risk factors for sarcopenia [2–4]. Currently, the pathogenesis of sarcopenia is unclear. However, sarcopenia may be related to genetics, nutritional deficiencies, neuromuscular function, hormones, autophagy, mitochondrial abnormalities, and gut flora [5, 6]. Sarcopenia not only increases the fall rate, disability rate, hospitalization rate, surgical complication rate, and even mortality but also affects the occurrence, development, and prognosis of various diseases [7]. However, research on sarcopenia is currently in the exploratory stage, and the association of preoperative sarcopenia with surgery is unclear.

The mass and strength of skeletal muscles are affected by various factors, such as age, gender, underlying diseases, dietary habits, and exercise, and can reflect the overall functional status [8]. In general, the prognosis of surgical patients is closely related to the functional status [9]. Patients with sarcopenia, as a special group, often exhibit low physical function, and they may be at an increased risk of postsurgical complications [10, 11]. Moreover, sarcopenia can lead to a decrease in skeletal muscle and the weakening of respiratory and swallowing muscles, thereby resulting in atelectasis, pneumonia, dysphagia, and malnutrition [12]. These aforementioned factors may increase postoperative complications and mortality, prolong hospital stay, affect quality of life, and increase health-care costs [13]. Therefore, surgeons and anesthesiologists should pay increased attention to patients with sarcopenia, as a potential high-risk group for adverse postoperative outcomes.

The influence of preoperative sarcopenia on the prognosis of major postoperative outcomes is unclear. The findings of previous studies on the association of sarcopenia with adverse postoperative outcomes and mortality are conflicting, and studies with an adequate sample size, clear definition of sarcopenia, and satisfactory results are scant [13–16]. Therefore, large-scale clinical research should be conducted on the prognosis of postoperative outcomes in patients with sarcopenia by utilizing a real-world database. Therefore, we conducted a comparative study through PSM to estimate the effects of preoperative sarcopenia on the outcomes of elective surgery.

## Patients and methods

### Data sources

We used data from January 2016 to December 2019 from Taiwan’s National Health Insurance (NHI) Research Database (NHIRD). The follow-up duration was from the index date to December 31, 2020. The NHIRD contains registration files and the original claims data of all NHI beneficiaries (i.e., approximately 27.38 million individuals). All NHIRD data, which are encrypted to ensure beneficiaries’ privacy, include detailed outpatient and inpatient claims information such as patient identification number; birth date; sex; diagnostic codes according to *International Classification of Diseases, Ninth Revision, Clinical Modification* (*ICD-9-CM*) and *International Classification of Diseases, Tenth Revision, Clinical Modification* (*ICD-10-CM*); treatment information; medical costs; dates of hospital admission and discharge; and date of death [17–21]. All data sets were interlinked using patient identification numbers. Our protocols were reviewed protocols were reviewed and approved by the Institutional Review Board of Tzu-Chi Medical Foundation (IRB109-015-B).

### Ethics

I would like to clarify that informed consent was waived in our study. This decision was made in accordance with the Personal Information Protection Act, as the data sets used in our research fall under its provisions.

### Participant selection

We selected 254,222 elderly patients aged ≥ 60 years who underwent major elective inpatient surgery; these old-age patients required general, epidural, or spinal anesthesia and hospitalization for more than 1 day between 2016 and 2019 in Taiwan. The evaluation of CT scans was limited to the 12-month preoperative period. Among the selected patients, 12,158 patients with a diagnosis of sarcopenia and 242,067 without a diagnosis of sarcopenia were categorized into the sarcopenia and nonsarcopenia groups, respectively (Supplemental Table 1). Before 2016, because of a lack of consensus regarding the definition of sarcopenia, a variety of diagnostic criteria were used [22]. In October 2016, the US Centers for Disease Control and Prevention formally recognized sarcopenia as a disease and coded it as M62.84 in ICD-10-CM [23]. We defined sarcopenia according the ICD-10-CM code after 2016 [17]. At least two claims for patients with a principal diagnosis of sarcopenia within the 12-month preoperative period were defined as the criteria for sarcopenia diagnosis. In Taiwan, sarcopenia was coded according to the results of a previous Taiwan study [24]; sarcopenia was defined as the skeletal muscle mass index (SMI) of 2 standard deviations or more below the normal sex-specific mean values for young persons. The date of onset of diabetes was regarded as the index date. The SMI was calculated using the following formula: SMI = L3 skeletal muscle cross-sectional area (cm^2^)/height^2^(m^2^), which was measured from computed tomography images [25].

### PSM and covariates

After adjustment for confounders, we used a multivariate logistic regression model to assess 30-day or 90-day postoperative complications onset from the index date (surgical date) in patients with and without preoperative sarcopenia. To reduce the effects of potential confounders when comparing adverse postoperative outcomes between the sarcopenia and nonsarcopenia groups, we matched all patients through PSM according to the following variables: age, sex, income levels, urbanization, coexisting medical conditions, hospital level, type of anesthesia, ASA score, and surgical type (Table 1). We matched the cohorts at a ratio of 1:4 using a greedy matching method, and the covariates were matched within a caliper with a propensity score of 0.2 [26]. Comorbidities were determined according to ICD-9-CM codes in the main diagnosis records of inpatients or were defined if the number of outpatient visits was ≥ 2 within 1 year. Comorbidities that occurred 2 years before the index date were included in this study.

**Table 1:** Characteristics of Surgical Patients With and Without Preoperative Sarcopenia (After Propensity Scores Were Matched)

	Nonsarcopenia		Sarcopenia		
	*N* = 48,632		*N* = 12,158		*P* value
**Age** (mean ± SD)	74.65 ± 16.94		74.65 ± 16.94		0.9999
Age groups, years old					0.9999
60–65	4,128	8.49%	1,032	8.49%	
66–70	6,256	12.86%	1,564	12.86%	
71–75	8,284	17.03%	2,071	17.03%	
76–80	10,152	20.88%	2,538	20.88%	
81–85	9,144	18.80%	2,286	18.80%	
> 85	10,668	21.94%	2,667	21.94%	
**Sex**					0.9999
Female	29,232	60.11%	7,308	60.11%	
Male	19,400	39.89%	4,850	39.89%	
**Income level (NTD)**					0.9999
Low income	512	1.05%	128	1.05%	
≤20,000	35,916	73.85%	8,979	73.85%	
20,001–30,000	5,688	11.70%	1,422	11.70%	
>30,000	6,516	13.40%	1,629	13.40%	
**Urbanization**					0.9999
Rural	10,984	22.59%	2,746	22.59%	
Urban	37,648	77.41%	9,412	77.41%	
**Coexisting medical conditions**					
Hypertension	17,736	36.47%	4,434	36.47%	0.9999
COPD	7,684	15.80%	1,921	15.80%	0.9999
Rheumatoid arthritis	1,596	3.28%	399	3.28%	0.9999
Diabetes	8,812	18.12%	2,203	18.12%	0.9999
Hyperlipidemia	11,672	24.00%	2,918	24.00%	0.9999
Renal dialysis	2,828	5.82%	707	5.82%	0.9999
Osteoporosis	4,220	8.68%	1,055	8.68%	0.9999
Stroke	4,860	9.99%	1,215	9.99%	0.9999
Congestive heart failure	2,220	4.56%	555	4.56%	0.9999
Peripheral vascular disease	1,252	2.57%	313	2.57%	0.9999
Hypothyroidism	504	1.04%	126	1.04%	0.9999
Myocardial infarction	324	0.67%	81	0.67%	0.9999
Acute renal failure	364	0.75%	91	0.75%	0.9999
**Hospital levels**					0.9999
Medical centers	34,688	71.13%	8,672	71.13%	
Non-medical centers	13,944	28,67%	3,486	28,67%	
**Types of anesthesia**					0.9999
General	39,180	80.66%	9,795	80.66%	
Epidural or spinal	9,452	19.44%	2,363	19.44%	
**ASA scores**					
I	16,829	34.59%	4,205	34.59%	0.9999
II	7,952	16.35%	1,988	16.35%	0.9999
III	21,12	43.41%	5,278	43.41%	0.9999
IV	2,748	5.65%	687	5.65%	0.9999
**Surgical types**					
Skin	812	1.67%	203	1.67%	0.9999
Breast	908	1.87%	227	1.87%	0.9999
Musculoskeletal	14,076	28.94%	3,519	28.94%	0.9999
Respiratory	2096	4.31%	524	4.31%	0.9999
Cardiovascular	1844	3.79%	461	3.79%	0.9999
Digestive	10,664	21.93%	2,666	21.93%	0.9999
Kidney, ureter, bladder	11,240	23.11%	2,810	23.11%	0.9999
Neurosurgery	6000	12.34%	1,500	12.34%	0.9999
Eye	404	0.83%	101	0.83%	0.9999

*Abbreviations* *ASA* American Society of Anesthesiology; *SD* standard deviation; *y* years-old; *NTD* New Taiwan Dollars; *N* number; *COPD* chronic obstructive pulmonary disease

Continuous variables are presented as means ± standard deviations where appropriate. A PSM ratio of 1:4 was used for the preoperative sarcopenia and nonsarcopenia groups; this ratio is commonly used to select controls with identical background covariates to minimize the differences among participants (we considered using controls based on previous studies) [27–32]. A multivariate logistic regression model was used to analyze postoperative complications in surgical patients with and without preoperative sarcopenia [33]. Using multivariate logistic regression analysis, we calculated odd ratios (ORs) with 95% confidence intervals (CIs) to determine whether preoperative sarcopenia is a potential independent predictor of 30-day or 90-day postoperative complications.

### Outcome measures

Eight major postoperative complications were monitored [28].: acute myocardial infarction, acute renal failure, deep-wound infection pneumonia, postoperative bleeding, pulmonary embolism, septicemia, and stroke. In our study, we utilized the Clavien Dindo classification system to categorize and describe postoperative complications, focusing on Grade 2 or higher complications [34]. The primary outcomes of this study were the complications and subsequent overall in-hospital mortality within 30 days after index surgery [27–32]. Studies have suggested that events recorded within 90 days of surgery are also postoperative complications [35–39].

### Data analysis

We used χ^2^ tests to analyze the descriptive parameters of demographic characteristics and coexisting medical conditions in the comparison of postoperative complications and death rates of patients with and without preoperative sarcopenia. Continuous variables were analyzed using *t* tests to compare the differences between patients with sarcopenia and controls. Multivariate logistic regression was used to analyze 30-day and 90-day postoperative complications and mortality between surgical patients with or without sarcopenia through the calculation of the adjusted ORs (aORs) with 95% CIs, with adjustment for age, sex, income level, urbanization, coexisting medical conditions, hospital level, type of anesthesia, ASA score, and surgical type. The logistic regression model’s goodness-of-fit was comprehensively assessed using both the Hosmer-Lemeshow test and the Omnibus test. The Hosmer-Lemeshow test evaluated the agreement between observed and expected outcomes across subgroups, while the Omnibus test assessed the overall significance of the model. These model fit assessments, including the results of the Hosmer-Lemeshow test and Omnibus test, ensured the validity and appropriateness of our logistic regression model. The statistical analysis software program V.9.4 (SAS Institute, Cary, North Carolina, USA) was used for data analyses; the differences between the groups were considered significant if two-sided *P* values were < 0.05.

## Results

### Study cohort

The data of 60,790 surgical patients (i.e., 12,158 and 48,632 in the sarcopenia and nonsarcopenia groups, respectively) were included in this study for further analysis; their characteristics are listed in Table 1. After frequency matching, the between-group differences in age, sex, income levels, urbanization, coexisting medical conditions, hospital levels, types of anesthesia, ASA scores, and surgical types were nonsignificant. The confounders (before matching) in the sarcopenia group significantly differed from those in the nonsarcopenia group (*p* < .001; Supplemental Table 1). Compared with the nonsarcopenia group, the sarcopenia group had more individuals who were older, were female, had a low income, were rural residents, had more coexisting medical conditions, received surgery at medical centers, and received general anesthesia (Supplemental Table 1).

### 30-day or 90-day adverse postoperative outcomes

Patients with sarcopenia exhibited higher rates of 30-day postoperative complications, including postoperative pneumonia (1.18% vs. 0.93%; *P* = .0134), postoperative bleeding (0.09% vs. 0.04%; *P* = .0420), septicemia (0.76% vs. 0.53%; *P* = .0028), and overall complications (6.85% vs. 5.62%; *P* = .0162; Table 2). The 30-day postoperative mortality rates for surgical patients with and without sarcopenia were 1.21% and 0.94%, respectively (*P* = .0085). Moreover, patients with sarcopenia exhibited higher rates of 90-day postoperative complications, including postoperative pneumonia (2.23% vs. 1.63%; *P* < .0001), postoperative bleeding (0.13% vs. 0.07%; *P* = .0267), septicemia (1.50% vs. 0.92%; *P* < .0001), and overall complications (8.85% vs. 7.95%; *P* = .0111). The 90-day postoperative mortality rates for surgical patients with and without sarcopenia were 2.08% and 1.38%, respectively (*P* < .0001).

**Table 2:** Adverse postoperative outcomes among matched surgical patients with and without preoperative sarcopenia

	Nonsarcopenia		Sarcopenia		
Outcomes	*N* = 48,632		*N* = 12,158		*P* value
**Postoperative complications (within 30 d)**	N	%	N	%	
Acute myocardial infarction	66	0.14%	21	0.17%	.3342
Acute renal failure	100	0.21%	25	0.21%	.9999
Deep wound infection	187	0.38%	53	0.44%	.4188
Pneumonia	452	0.93%	143	1.18%	.0134
Pulmonary embolism	91	0.19%	19	0.16%	.4741
Postoperative bleeding	21	0.04%	11	0.09%	.0420
Septicemia	260	0.53%	93	0.76%	.0028
Stroke	1842	3.79%	468	3.85%	.7503
Any of the above	2734	5.62%	833	6.85%	.0162
30-d postoperative mortality	459	0.94%	147	1.21%	.0085
**Postoperative complications (within 90 d)**					
Acute myocardial infarction	105	0.22%	36	0.30%	.1002
Acute renal failure	152	0.31%	44	0.36%	.3906
Deep wound infection	261	0.54%	66	0.54%	.9337
Pneumonia	791	1.63%	271	2.23%	< .0001
Pulmonary embolism	111	0.23%	21	0.17%	.2395
Postoperative bleeding	33	0.07%	16	0.13%	.0267
Septicemia	447	0.92%	182	1.50%	< .0001
Stroke	2538	5.22%	665	5.47%	.2681
Any of the above	3865	7.95%	1,076	8.85%	.0011
90-d postoperative mortality	669	1.38%	253	2.08%	< 0.0001

*Abbreviations* *N* number

### Adjusted ORs and 95% CIs for 30-day or 90-day adverse postoperative outcomes

After adjustment for age, sex, income levels, urbanization, coexisting medical conditions, hospital levels, types of anesthesia, ASA scores, and surgical types, our multivariate logistic regression analyses revealed that surgical patients with preoperative sarcopenia were at significantly higher risk of 30-day postoperative mortality (aOR = 1.25; 95% CI 1.03 to 1.52) and 30-day major complications, including postoperative pneumonia (aOR = 1.15; 95% CI = 1.00-1.40), postoperative bleeding (aOR = 2.18; 95% CI = 1.04–4.57), septicemia (aOR = 1.31; 95% CI = 1.03–1.66), and overall complications (aOR = 1.13; 95% CI = 1.00-1.46). In addition, surgical patients with sarcopenia were at significantly higher risk of 90-day postoperative mortality (aOR = 1.50; 95% CI = 1.29–1.74) and 90-day major complications, including postoperative pneumonia (aOR = 1.27; 95% CI = 1.10–1.47), postoperative bleeding (aOR = 1.90; 95% CI = 1.04–3.48), septicemia (aOR = 1.52; 95% CI = 1.28–1.82), and overall complications (aOR = 1.24; 95% CI = 1.08–1.42; Table 3).

**Table 3:** Adjusted ORs and 95% CIs for adverse postoperative outcomes associated with preoperative sarcopenia

	Control	Sarcopenia			
Outcomes	*N* = 48,632	*N* = 12,158	aOR^*^	(95% CI)	*P* value
**Postoperative complications (within 30 d)**					
Acute myocardial infarction	0.14%	0.17%	1.16	(0.68, 1.98)	.5850
Acute renal failure	0.21%	0.21%	0.92	(0.52, 1.29)	.3931
Deep wound infection	0.38%	0.44%	1.11	(0.81, 1.51)	.5223
Pneumonia	0.93%	1.18%	1.15	(1.00, 1.40)	.0492
Pulmonary embolism	0.19%	0.16%	0.88	(0.49, 1.34)	.4156
Postoperative bleeding	0.04%	0.09%	2.18	(1.04, 4.57)	.0393
Septicemia	0.53%	0.76%	1.31	(1.03, 1.66)	.0307
Stroke	3.79%	3.85%	0.91	(0.81, 1.03)	.1262
Any of the above	6.21%	6.85%	1.13	(1.00, 1.46)	.0438
30-d postoperative mortality	0.94%	1.21%	1.25	(1.03, 1.52)	.0212
**Postoperative complications (within 90 d)**					
Acute myocardial infarction	0.22%	0.30%	1.19	(0.79, 1.79)	.4037
Acute renal failure	0.31%	0.36%	0.97	(0.68, 1.36)	.8433
Deep wound infection	0.54%	0.54%	1.01	(0.75, 1.3)	.9447
Pneumonia	1.63%	2.23%	1.27	(1.10, 1.47)	.0011
Pulmonary embolism	0.23%	0.17%	0.75	(0.47, 1.2)	.2304
Postoperative bleeding	0.07%	0.13%	1.90	(1.04, 3.48)	.0373
Septicemia	0.92%	1.50%	1.52	(1.28, 1.82)	< .0001
Stroke	5.22%	5.47%	0.92	(0.83, 1.03)	.1414
Any of the above	7.95%	8.85%	1.24	(1.08, 1.42)	.0018
90-d postoperative mortality	1.38%	2.08%	1.50	(1.29, 1.74)	< 0.0001

*Abbreviations* *OR* odds ratio; *aOR* adjusted odds ratio; *CI* confidence interval; *N>* number

*All covariates mentioned in Table 1 were adjusted

### Kaplan–Meier 30-day or 90-day postoperative mortality and complications

**Fig. 1:**
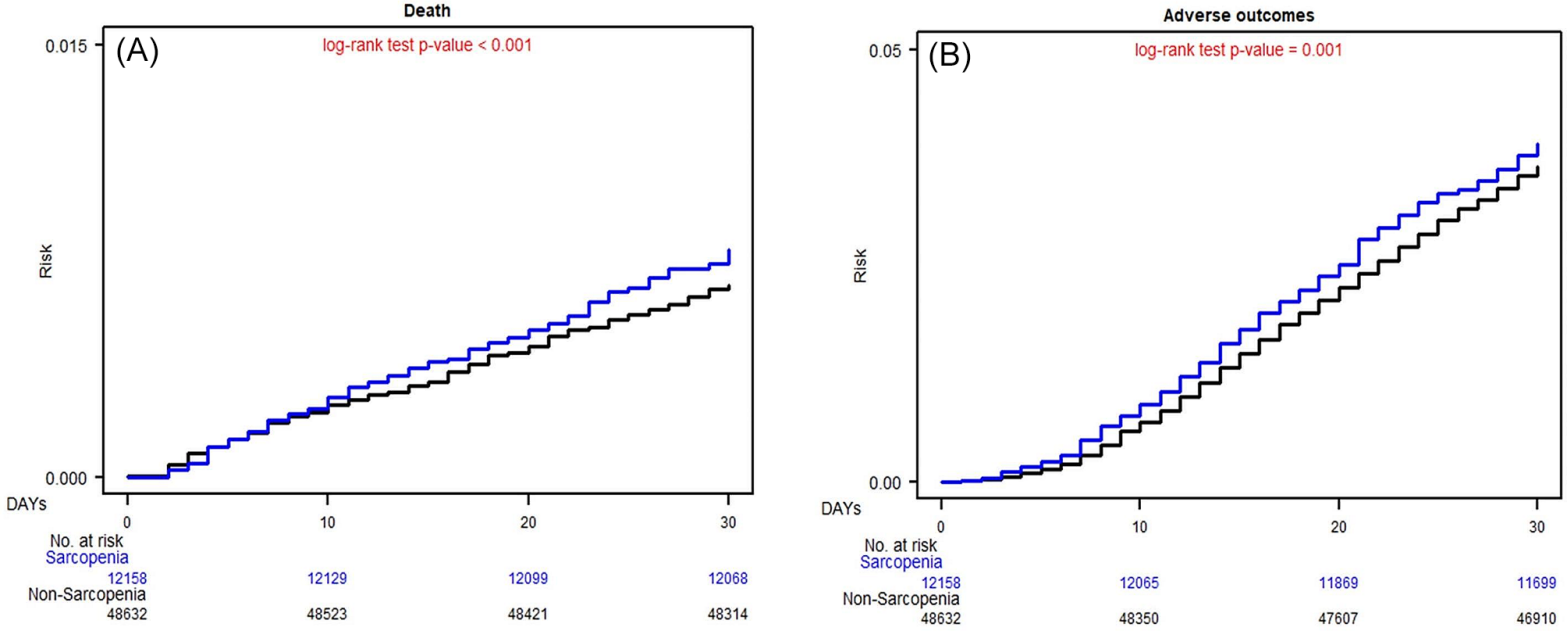
Kaplan–Meier Estimates of 30-d Postoperative Mortality and 30-d Postoperative Complications Among Surgical Patients With and Without Sarcopenia. (**A**) 30-d Postoperative Mortality; (**B**) 30-d Overall Postoperative Complications

**Fig. 2:**
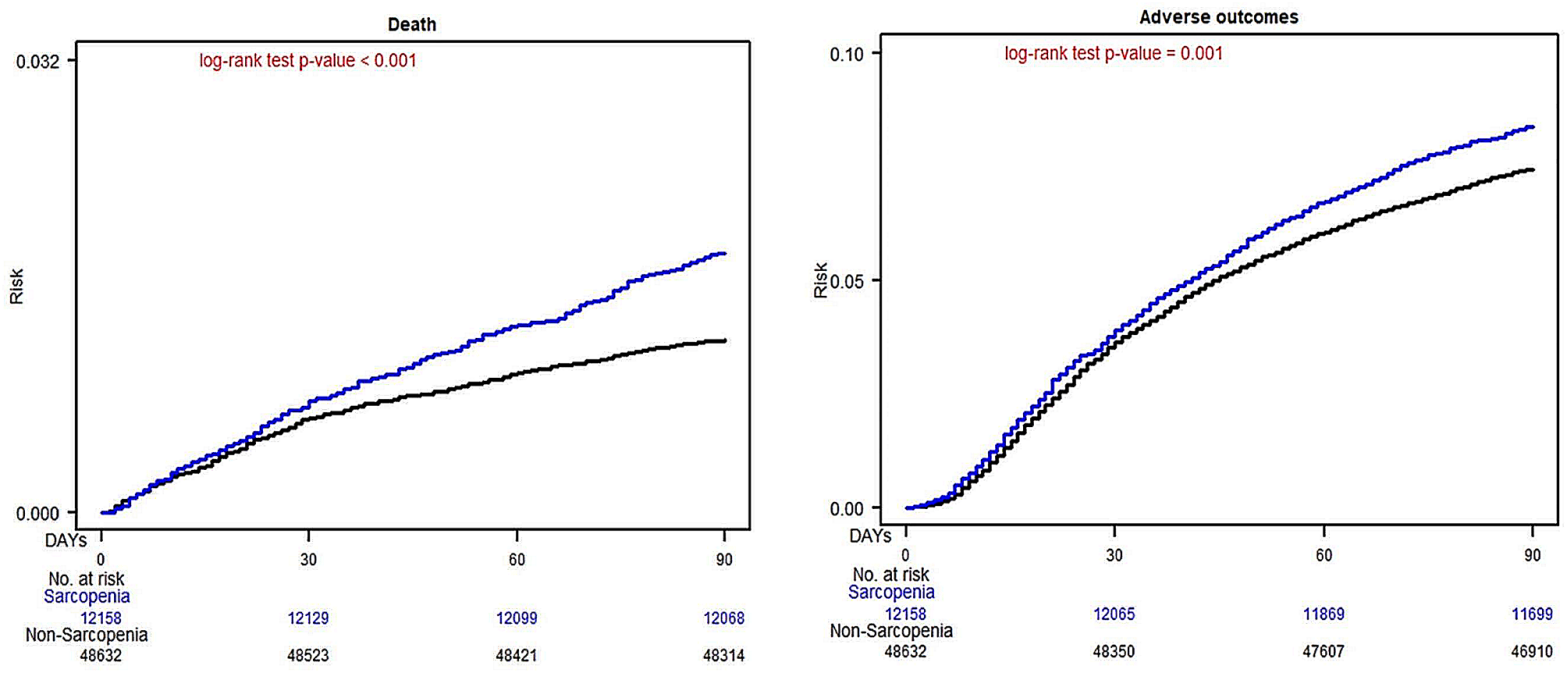
Kaplan–Meier Estimates of 90-d Postoperative Mortality and 90-d Postoperative Complications Among Surgical Patients With and Without Sarcopenia. (**A**) 90-d Postoperative Mortality; (**B**) 90-d Overall Postoperative Complications

Figure 1 illustrates the cumulative risks of 30-day or 90-day postoperative mortality and complications in matched patients with and without sarcopenia. The cumulative 30-day postoperative mortality was significantly higher in the sarcopenia group than in the nonsarcopenia group (*P* < .0001; Fig. 1A), and the cumulative overall 30-day postoperative complications were significantly higher in the sarcopenia group than in the nonsarcopenia group (*P* < .0001; Fig. 1B). Moreover, the Kaplan–Meier curves revealed that the cumulative 90-day postoperative mortality and overall complications were significantly lower in the sarcopenia group than in the nonsarcopenia group (*P* < .0001; Fig. 2).

## Discussion

Clinically, many patients with sarcopenia experience various challenges and severe complications of surgery [40]. However, the association of preoperative sarcopenia with adverse postoperative outcomes and mortality is unclear [13–16]. To the best of our knowledge, the present study is the first and the largest PSM-based comparative study on 30-day and 90-day adverse postoperative outcomes and mortality among patients with and without preoperative sarcopenia. This retrospective, real-world data-derived, population-based PSM cohort study revealed that preoperative existing sarcopenia is an independent risk factor for 30-day and 90-day adverse postoperative outcomes, such as postoperative pneumonia, postoperative bleeding, and septicemia, and preoperative sarcopenia is associated with increased 30-day and 90-day postoperative mortality among patients receiving major surgery. Multivariate logistic regression analyses revealed that surgical patients with preoperative sarcopenia were at significantly higher risk of 30-day postoperative mortality (aOR = 1.25; 95% CI = 1.03–1.52) and 30-day major complications, including postoperative pneumonia (aOR = 1.15; 95% CI = 1.00-1.40), postoperative bleeding (aOR = 2.18; 95% CI = 1.04–4.57), septicemia (aOR = 1.31; 95% CI = 1.03–1.66), and overall complications (aOR = 1.13; 95% CI = 1.00-1.46). In addition, surgical patients with sarcopenia were at significantly higher risk of 90-day postoperative mortality (aOR = 1.50; 95% CI = 1.29–1.74) and 90-day major complications, including postoperative pneumonia (aOR = 1.27; 95% CI = 1.10–1.47), postoperative bleeding (aOR = 1.90; 95% CI = 1.04–3.48), septicemia (aOR = 1.52; 95% CI = 1.28–1.82), and overall complications (aOR = 1.24; 95% CI = 1.08–1.42). Our results provide valuable comprehensive information on postoperative complications, especially postoperative pneumonia, postoperative bleeding, and postoperative septicemia, among patients with sarcopenia receiving surgery. Establishing a comprehensive protocol to prevent the aforementioned postoperative complications that contribute to surgical mortality can be valuable to future research.

Several reports have indicated similar outcomes [41–44], suggesting an association between preoperative sarcopenia and increased postoperative complications [41–44]. In our extensive investigation, we explored the impact of preoperative sarcopenia on postoperative outcomes within a cohort of 254,222 elderly patients (≥ 60 years) undergoing major elective inpatient surgery in Taiwan between 2016 and 2019. Utilizing Taiwan’s NHIRD, which includes detailed claims data for approximately 27.38 million individuals, our study stands out for its large cohort size, facilitating robust statistical analyses. Unlike earlier studies that employed diverse diagnostic criteria for sarcopenia, our research defined sarcopenia post-2016 using the ICD-10-CM code M62.84 and incorporated specific criteria based on the SMI derived from CT scans. This nuanced approach allows for a more precise identification of sarcopenic patients. Furthermore, our study employed PSM and multivariate logistic regression to account for potential confounders, ensuring a meticulous analysis of 30-day and 90-day postoperative complications. The investigation of eight major complications, including acute myocardial infarction, acute renal failure, deep-wound infection pneumonia, postoperative bleeding, pulmonary embolism, septicemia, and stroke, adds granularity to the understanding of the outcomes. Furthermore, the concentration on individuals aged 60 and above, along with the meticulous matching of different surgical procedures through PSM, distinguishes our approach. Unlike previous studies primarily focusing on specific surgical types, such as cardiac or abdominal surgery [43, 44], our research expands the scope and enhances external validity due to a larger and more diverse sample [41–44]. Consequently, our conclusions elucidate a higher incidence of surgical complications among elderly individuals with sarcopenia, providing unique insights beyond the existing literature. Importantly, we extend our analysis beyond the conventional 30-day acute complications to include 90-day subacute complications, a novel contribution to the field. This study significantly advances our understanding of the intricate relationship between sarcopenia and postoperative outcomes, making a substantial and novel addition to the existing literature [41–44]. Our investigation not only scrutinizes acute complications within the conventional 30-day timeframe but extends its analysis to encompass subacute complications occurring within 90 days—a novel aspect absent in prior literature [41–44]. This extended timeframe provides a more comprehensive understanding of the postoperative complications associated with sarcopenia in the elderly, offering unique insights and a substantial addition to the existing literature.

The primary endpoints of our study were centered on complications and overall in-hospital mortality within 30 days following the index surgery, as supported by relevant literature [27–32]. Recognizing that postoperative events extend beyond the traditional 30-day window, recent studies have advocated for an extended observation period of 90 days to capture a more comprehensive spectrum of postoperative complications [35–39]. Specifically, the 90-day postoperative mortality metric has gained prominence as a robust measure of surgical quality, particularly for procedures involving the digestive tract or the head and neck [35–39]. Given the evolving understanding of the prolonged impact of surgery, we designated 90-day postoperative complications as a primary outcome in our study. This timeframe allows for a nuanced assessment of acute and subacute surgical complications, providing a more comprehensive perspective on patient outcomes. To maintain consistency with established practices and enhance comparability with prior research, we adhered to the definition of 30-day and 90-day in-hospital postoperative mortality as utilized in previous studies [27, 29–32, 35–39]. Consequently, patients who succumbed on the 91st day or later post-hospitalization were considered alive in our study, and those who died outside the hospital within 90 days were not included in the mortality outcome. In conclusion, the choice of assessing 30- and 90-day postoperative complications aligns with contemporary views on capturing the continuum of surgical outcomes, offering a more nuanced understanding of acute and subacute complications associated with the procedures under investigation.

A patient with sarcopenia receiving elective surgery is a patient with certain systemic imbalances and a worse biological reserve, which may contribute to a poor postoperative prognosis; this finding is consistent with a previous study finding [45]. Moreover, many risk factors are closely related to sarcopenia, for example, increasing age, malnutrition, alcoholism, smoking, insomnia, and chronic diseases, which often affect surgical prognosis and cause postoperative death [46–48]. Nevertheless, after matching for age, sex, income level, urbanization, coexisting medical conditions, hospital level, type of anesthesia, ASA score, and surgical type, sarcopenia is still an independent risk factor for 30-day and 90-day postoperative pneumonia, bleeding, septicemia, overall surgical complications, and mortality (Tables 2 and 3). In addition, not only the ORs of 30-day surgical complications and mortality but also the aORs of 90-day surgical complications and mortality were highly significant (Table 3; Fig. 2). The subacute surgical complications (90 d) may also be critical in patients with sarcopenia receiving surgery. Based on the outcomes of 30-day and 90-day surgical complications, patients with sarcopenia experience not only acute surgical complications but also subacute surgical complications after receiving elective surgery. The adverse outcomes of surgery in patients with sarcopenia do not alleviate even 30 days after surgery (Figs. 1 and 2).

The influence of sarcopenia on the prognosis of surgery is still unclear because of the small sample size, different definitions of sarcopenia, and different surgical types [10, 11, 13–16, 49–55]. Thus far, no study with sufficient sample size or with appropriate matching has investigated the association of sarcopenia with 30-day and 90-day surgical complications and mortality after elective surgery. To the best of our knowledge, this is the first study to use the latest definition of sarcopenia based on ICD-10 and to demonstrate 30-day and 90-day adverse outcomes of surgery in patients with sarcopenia and nonsarcopenia receiving elective surgery. According to our literature review, studies have not compared 30-day and 90-day surgical complications between sarcopenia and nonsarcopenia groups. Our study is the first to demonstrate that patients with sarcopenia exhibited significantly increased rates of 30-day and 90-day adverse outcomes, including postoperative pneumonia, bleeding, septicemia, and mortality after elective surgery.

Among various adverse outcomes after major surgery, postoperative septicemia is one of the key factors leading to the death of patients with sarcopenia [56–59]. Our findings indicated that the incidence of 30-day and 90-day postoperative septicemia was significantly higher among patients with sarcopenia than among those without sarcopenia (Tables 2 and 3). The high incidence of postoperative septicemia in sarcopenia patients receiving surgery can be attributed to low immunity compared with that of nonsarcopenia patients [60, 61]. Muscle fibers can produce cytokines and interleukins, inhibiting the secretion of tumor necrosis factor and mediating insulin resistance [62, 63]. Sarcopenia reduces cellular immune function, increases the level of proinflammatory factors, and increases the possibility of infection in the body [60, 61]. Moreover, the level of glutamine, which is an activator of lymphocytes and monocytes, is significantly reduced in patients with sarcopenia, thereby partly weakening their immunity [64]. Studies have suggested that sarcopenia may be one of the predictors of infection after colon cancer surgery [11].

Decreased immunity in patients with sarcopenia not only increases the possibility of postoperative infection but also increases the incidence of postoperative pneumonia [60, 61, 65]. In addition, patients with sarcopenia receiving surgery may experience difficulty in sputum removal and a high incidence of choking or aspiration pneumonia due to muscular weakness [66, 67]. Soma et al. reported that preoperative sarcopenia in patients with esophageal cancer increases the risk of postoperative respiratory disease [68]; this is consistent with our findings. The increase in postoperative pneumonia may be related to the decrease in skeletal muscle mass and the weakening of respiratory and swallowing muscles in patients with sarcopenia [66, 67]. Jain et al. demonstrated that short-term resistance exercise training and protein supplementation before surgery can increase the mass and strength of skeletal muscles and reduce fat content, thereby improving immunity and reducing the incidence of postoperative pulmonary complications [69]. Therefore, preoperative sarcopenia, swallowing function, and respiratory muscle training should be improved before elective surgery to decrease the incidence of postoperative pneumonia, other surgical complications, and mortality.

Our study discovered that patients with sarcopenia had a significantly increased incidence of 30-day and 90-day postoperative bleeding. To the best of our knowledge, no study has reported that sarcopenia directly alters the coagulation system; however, large retrospective studies have demonstrated that malnutrition can lead to postoperative bleeding in patients undergoing colorectal resection and pancreatic surgery, increase the risks of respiratory failure or infection, and even increase the mortality rate of patients [70, 71].

The present study used data from the NHIRD, which reliably records the detailed medical information of Taiwanese patients, and this database has been used in many high-quality studies [17, 20, 21, 27, 28]. Furthermore, a large PSM-based design was employed in the comparative study to maintain balance among the confounders of the case and control groups—all in the absence of bias (Table 1). However, this study has a few limitations. First, PSM cannot control factors that are not accounted for in the model, and it is predicated on an explicit selection bias of the factors that can be matched. Second, because all patients were enrolled from an Asian population, the corresponding ethnic susceptibilities in non-Asian populations are unclear. However, no significant differences in the postoperative adverse outcomes and mortality have been reported between Asian and non-Asian populations; the results should be cautiously extrapolated to non-Asian populations. Third, another limitation of this study pertains to the diagnosis of sarcopenia, which relies solely on the availability of the ICD code. Consequently, if an individual is unintentionally omitted from receiving the sarcopenia code, they are categorized as not having sarcopenia. We acknowledge that this approach may result in potential underestimation. Recognizing this limitation, we emphasize the importance of conducting further prospective studies to yield more accurate and comprehensive results in this area.

## Conclusions

We demonstrated that sarcopenia is an independent risk factor for 30-day and 90-day adverse postoperative outcomes such as postoperative pneumonia, bleeding, septicemia, and mortality after elective surgery. Therefore, preoperative sarcopenia, swallowing function, and respiratory muscle training should be corrected before elective surgery to reduce the incidence of postoperative complications that contribute to the decrease in surgical mortality.

### Electronic supplementary material

Below is the link to the electronic supplementary material.

Supplementary Material 1

## Data Availability

The datasets essential for supporting the conclusions of this study are provided within the manuscript and its supplementary files.

## Abbreviations

OR: Odds ratio
aOR: Adjusted odds ratio
CI: Confidence interval
ICD-9-CM: International Classification of Diseases, Ninth Revision, Clinical Modification
ICD-10-CM: International Classification of Diseases, Tenth Revision, Clinical Modification
PSM: Propensity score matching
NHIRD: National Health Insurance Research Database
ASA: American Society of Anesthesiology
SD: Standard deviation
SMD: Standardized mean difference
IQR: Interquartile range
y: Years-old
NHI: National Health Insurance
SMI: Skeletal muscle mass index
