# Supplementary material for: Adverse postoperative outcomes in elderly patients with sarcopenia
Source: BMC Geriatr. 2024 Jun 27;24:561. doi: 10.1186/s12877-024-05066-2 (PMC11212269; doi:10.1186/s12877-024-05066-2)
Supplement: Supplementary file 1 — Supplementary Material 1 [file 12877_2024_5066_MOESM1_ESM.pdf]

Supplemental Table 1. Characteristics of Surgical Patients With and Without Preoperative Sarcopenia (Before Propensity Scores Were Matched)

	Nonsarcopenia		Sarcopenia		
	N = 242,067		N = 12,158		*P* value
**Age** (mean ± SD)	47.26 ± 17.44		54.65 ± 16.94		<.0001
Age groups					<.0001
20 y < Age ≤ 30 y	44,267	18.29%	1,032	8.49%	
30 y < Age ≤ 40 y	50,771	20.97%	1,564	12.86%	
40 y < Age ≤ 50 y	45,673	18.87%	2,071	17.03%	
50 y < Age ≤ 60 y	37,648	15.55%	2,538	20.88%	
60 y < Age ≤ 70 y	30,371	12.55%	2,286	18.80%	
Age > 70 y	33,337	13.77%	2,667	21.94%	
**Sex**					<.0001
Female	137,830	56.94%	7308	60.11%	
Male	104,237	43.06%	4850	39.89%	
**Income levels (NTD)**					<.0001
Low income	2,542	1.05%	128	1.05%	
≤20000	152,140	62.85%	8979	73.85%	
20001–30000	42,646	17.62%	1422	11.70%	
>30000	44,739	18.48%	1629	13.40%	
**Urbanization**					<.0001
Rural	45,990	19.00%	2,746	22.59%	
Urban	196,077	81.00%	9,412	77.41%	
**Coexisting medical conditions**					
Hypertension	50,914	21.03%	4434	36.47%	<.0001
COPD	22,420	9.26%	1921	15.80%	<.0001
Rheumatoid arthritis	3,360	1.39%	399	3.28%	<.0001
Diabetes	26,092	10.78%	2203	18.12%	<.0001
Hyperlipidemia	28,455	11.76%	2918	24.00%	<.0001
Renal dialysis	7,689	3.18%	707	5.82%	<.0001
Osteoporosis	9,030	3.73%	1055	8.68%	<.0001
Stroke	13,255	5.48%	1215	9.99%	<.0001
Congestive heart failure	5,673	2.34%	555	4.56%	<.0001
Peripheral vascular disease	2,320	0.96%	313	2.57%	<.0001
Hypothyroidism	1204	0.50%	126	1.04%	<.0001

Myocardial infarction	1063	0.44%	81	0.67%	<.0001
Acute renal failure	806	0.33%	91	0.75%	<.0001
**Hospital levels**					<.0001
Medical centers	160,829	66.44%	8,672	71.13%	
Non-medical centers	81,238	33.56%	3,486	28,67%	
**Types of anesthesia**					<.0001
General	177,798	73.45%	9,795	80.66%	
Epidural or spinal	64,269	26.55%	2,363	19.44%	
**ASA scores**					
I	148,006	61.14%	4,205	34.59%	<.0001
II	29,045	12.00%	1,988	16.35%	<.0001
III	57,107	23.59%	5,278	43.41%	<.0001
IV	7,909	3.27%	687	5.65%	<.0001
**Surgical types**					<.0001
Skin	5,044	2.08%	203	1.67%	<.0001
Breast	3,980	1.64%	227	1.87%	.0239
Musculoskeletal	59,410	24.54%	3519	28.94%	<.0001
Respiratory	9,349	3.86%	524	4.31%	<.0001
Cardiovascular	7,283	3.01%	461	3.79%	<.0001
Digestive	54,993	22.72%	2666	21.93%	.0387
Kidney, ureter, bladder	79,590	32.88%	2810	23.11%	<.0001
Neurosurgery	16,942	7.00%	1500	12.34%	<.0001
Eye	2,415	1.00%	101	0.83%	<.0001

Abbreviations: ASA, American Society of Anesthesiology; SD, standard deviation; y, years-old; NTD, New Taiwan Dollars, N, Number; COPD, chronic obstructive pulmonary disease.
